# Joint Action of a Pair of Rowers in a Race: Shared Experiences of Effectiveness Are Shaped by Interpersonal Mechanical States

**DOI:** 10.3389/fpsyg.2016.00720

**Published:** 2016-05-18

**Authors:** Mehdi R’Kiouak, Jacques Saury, Marc Durand, Jérôme Bourbousson

**Affiliations:** ^1^“Movement, Interactions, Performance" Laboratory (EA4334), University of NantesNantes, France; ^2^University of GenevaGeneva, Switzerland

**Keywords:** mixed method, enaction, interpersonal coordination, extrapersonal coordination, rowing, course of action, subjectivity-based sampling method

## Abstract

The purpose of this study was to understand how a single pair of expert individual rowers experienced their crew functioning in natural conditions when asked to practice a joint movement for the first time. To fulfill this objective, we conducted a field study of interpersonal coordination that combined phenomenological and mechanical data from a coxless pair activity, to analyze the dynamics of the (inter)subjective experience compared with the dynamics of the team coordination. Using an enactivist approach to social couplings, these heterogeneous data were combined to explore the salience (and accuracy) of individuals’ shared experiences of their joint action. First, we determined how each rower experienced the continuous crew functioning states (e.g., feelings of the boat’s glide). Second, the phenomenological data helped us to build several categories of oar strokes (i.e., cycles), experienced by the rowers as either detrimentally or effectively performed strokes. Third, the mechanical signatures that correlated with each phenomenological category were tracked at various level of organization (i.e., individual-, interpersonal-, and boat-levels). The results indicated that (a) the two rowers did not pay attention to their joint action during most of the cycles, (b) some cycles were simultaneously lived as a salient, meaningful experience of either a detrimental (*n* = 15 cycles) or an effective (*n* = 18 cycles) joint action, and (c) the mechanical signatures diverged across the delineated phenomenological categories, suggesting that the way in which the cycles were experienced emerged from the variance in some mechanical parameters (i.e., differences in peak force level and mean force). Notably, the mechanical measures that helped to explain differences within the phenomenological categories were found at the interpersonal level of analysis, thus suggesting an intentional inter-personal mode of regulation of their joint action. This result is further challenged and discussed in light of extra-personal regulation processes that might concurrently explain why participants did not make an extensive salient experience of their joint action. We conclude that attempts to combine phenomenological and mechanical data should be pursued to continue the research on how individuals regulate the effectiveness of their joint actions’ dynamics.

## Introduction

Joint action is a ubiquitous phenomenon underlying most daily activities, especially when interpersonal sensorimotor coupling is involved. Joint action has been abundantly investigated in human movement science using kinematics descriptions (Schmidt and O’Brien, 1997; Romero et al., 2012), and to a lesser extent by describing the embodied perceptive (and/or subjective) activities implied in its active regulation (Laroche et al., 2014). In the mainstream research on joint action, most studies that have involved the participants’ lived (i.e., subjective) experience of ongoing team coordination have been controlled by the experimental instructions given to the participants.

The first part of this stream of research has considered the role of the participant’s lived concerns by focusing on the intentional features (i.e., the participant’s explicit experience of regulating his behavior) underlying the regulatory mechanisms. Typically, pairs of participants were asked to coordinate their oscillating legs (alternately in phase and anti-phase patterns) and to actively/explicitly regulate the coordination so that the emergent states of synchrony/asynchrony perceived on the fly would remain stable overtime (Schmidt et al., 1990). This study has been compared to a companion one in which participants instead were asked to remain aware of their lived experience of comfort and to regulate their behavior accordingly. The comparison of both types of awareness showed that the degree of active perceptive regulation was a critical process that controlled the fluctuations and phase transitions within the emerging team coordination states. Such observations particularly illustrate how a change in the subjective regulation of the participants (i.e., being more or less active or/and explicit to them) might shape the biomechanical signatures of the ongoing joint action.

The second part of the research has focused on the unperceived aspects underpinning the dynamics of team coordination, which form the behavioral facet of the coordination that is meaningless to the participants. To illustrate, Schmidt and O’Brien (1997) asked participants, placed in pairs, to avoid synchronous oscillations while swinging a pendulum with their arms. They observed that the participants were able to prevent this coordination from occurring only in the absence of informational exchanges (i.e., not mutually visible). Otherwise, and despite the instruction of avoiding synchronization, a tendency to phase-lock emerged when the participants were informationally coupled (i.e., they were able to perceive each other’s moves). Such study highlights how implicit features (i.e., an absence of awareness of the emerging team coordination states) shape the action and perception loop underlying joint action. In doing so, this study questions both the way in which actors might be aware of their ongoing interaction, and the way in which explicit/meaningful regulation is shaped by processes similar to unintentional/unperceived coordination.

While some researchers call for investigating the lived experience of the actors as an important part of the joint action process itself (De Jaegher and Di Paolo, 2007), very few studies have considered the awareness and the sense-making activity of the actors as a valuable topic for research (Gallagher, 2009; De Jaegher et al., 2010; Froese and Di Paolo, 2010). Yet, empirical evidence has shown that with increasing expertise, actors are more likely to use lived experience to actively regulate the dynamics of the joint action (Schiavio and Høffding, 2015). In this light, thanks to their study of team rowing coordination in a natural setting, Lund et al. (2012) suggested that participants learned to coordinate by gradually and systematically adjusting their shared experiences over time. As claimed by Heath et al. (2002), such an active regulation by actors in organizational settings is enable by a skillful use of their lived experience to monitor the ongoing team coordination of which they are part. However, very little evidence has been provided of the salience and accuracy of such an online awareness in either human movement science or sports science. Together, these elements demonstrate that the way joint movement is experienced remains a neglected topic within joint action research.

A recent study carried out on the sport field selected rowing as a setting to describe how athletes experienced their activity and the accuracy of their awareness (Millar et al., 2015). While the study investigated coordination phenomena only at an intrapersonal scale, it gave insights into the role of the online lived experience of actors in regulating their action and perception dynamics. In particular, the study suggested that with increased expertise, the rowers are more likely to be aware of the ongoing changes within the performance states (i.e., change in boat speed), even more than their coaches are from their external point of view. This study thus illustrates how expert performers might be able reliably to live and account for their dynamical individual activity. However, it is still unknown whether expert individual performers exhibit the same salient awareness of their activity when involved in a joint action task. In this light, investigations of phenomenological phenomena are still needed in the research on joint action processes. Quite novel in the field, the present study was exploratory and described the systematic lived experience of participants regarding their joint movement. The study was conducted in a natural setting of rowing. By combining phenomenological data with behavioral data (i.e., mechanical measures) and by using an original methodological design, we aimed to discuss the ways in which humans actively manage their emerging experience of the team coordination states.

The present study was designed with respect for an enactivist approach to social couplings (De Jaegher and Di Paolo, 2007; Laroche et al., 2014) to address the extent to which actors had shared meaningful lived experiences through the joint movement behavioral states. By combining a phenomenological description of their activity with a behavioral description, we aimed to explore the accuracy of such experiences.

The enactivist view to social couplings assumes that joint action processes should be investigated by reconstructing the way in which individuals live in their own worlds that are mutually coupled. Such a joint sense-making activity is assumed precarious in that individuals sense-making activities shape and are shaped by the fluctuating dynamics of the behavioral facet of the coupling to which they are contributing. An enactivist approach to the analysis of joint action thus aims to describe how the behavioral facet of the social coupling needs to complement the (inter)subjective facet in which it is embedded. This framework aims to contribute to a paradigm shift in cognitive science (Varela, 1979), as the researchers present a non-representational frameworks in social cognition science (e.g., Varela, 1979; Varela et al., 1991). Instead of rejecting the subjectivity of participants (i.e., as in some of the non-representationalist views of cognition), the enactivist approach conceives it as a main component in the active regulation of the situated embodied activity. Thus, following a careful phenomenological framework (e.g., Theureau, 2003), the enactivist approach considers the “own world” of humans as the product of (a) the nature of their sensory apparatus that is genetically inherited, (b) the history of the actor/environment coupling (e.g., recurrent patterns of perception and action built during individual development), and (c) the way in which individual experiences his/her coupling with the environment in the moment (Thompson, 2011). This last assumption makes the situated experience lived by each of the performers the *sine qua non* condition for describing how their behaviors are systematically arranged into dynamic patterns in their real-time activity.

The present field study of joint action in a rowing crew combined the data from two alternative research traditions within activity analysis: the dynamics of the lived experience and the dynamics of the behavior. These data have been combined with a view to explore how individual lived experiences are tightly nested in the active regulation of joint action between two elite performers who have not been trained to row together. To explore the behavioral facet in which lived experiences are dynamically anchored, our starting point was to determine how each rower experienced the continuous coordination states during their race. Such phenomenological data helped to build several samples of oar strokes, differentially experienced. Grounded on such a subjectivity-based sampling method (Lutz et al., 2002), we then scrutinized the behavioral facet of the strokes by characterizing the specific behavioral signatures underlying the identified lived experiences, as captured at various levels of analysis. The following research questions drove the present study: (a) to what extent do individual coxless pair rowers report salient, meaningful lived experiences of their joint action effectiveness? (b) To what extent are these experiences similar across rowers? (c) Are distinct shared lived experiences of joint action effectiveness associated with distinct mechanical signatures? Finally, (d) to what extent do shared lived experiences of joint action effectiveness capture behavioral instances of expert team coordination?

## Materials and Methods

### Characteristics of the Setting under Study

The naturalistic conditions of rowing (i.e., on water) have been selected for investigating joint action and the related shared lived experiences of rowers. Team coordination has been shown to be one of the major performance factors in crews of two or more rowers (Wing and Woodburn, 1995; Hill, 2002; Smith and Draper, 2002; Baudouin and Hawkins, 2004). In such an interactive performance setting, rowers are mutually involved in a permanent real-time regulation of the emerging behavioral states of team coordination (Pinder et al., 2011). Much feedback is available for rowers during their race –they can feel their teammate’s oar blade enter the water through the boat movement, the boat’s roll, or their common propulsion, which makes this setting also particularly attractive for exploring the rowers’ lived experience (Millar et al., 2013). This abundance of feedbacks is likely to produce a rich amount of sense-making activity, although it may make it complex. Moreover, the existing mechanical capture systems allow the collection of a large amount of behavioral data in natural settings (i.e., on the water) at different levels of the social system: individual level (e.g., forces, angles measures; Ishiko, 1971; Schneider et al., 1978; Kleshnev, 2011); interpersonal level (e.g., time gaps in the entry into water of the rowers’ oar; Sève et al., 2013) and the boat’s level (e.g., boat speed; Hill, 2002; Baudouin and Hawkins, 2004). Such a setting offers a rich opportunity to advance the research on team coordination in general and on multi-level approaches of joint action in particular (Cooke et al., 2013; Kozlowski et al., 2013; Humphrey and Aime, 2014; Bourbousson et al., 2015).

### Participants and Procedure

**FIGURE 1:**
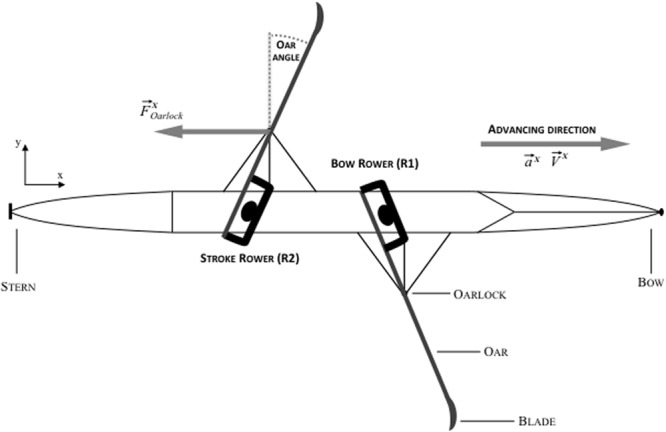
**Bird’s-eye view of a coxless pair.** The onboard measurement system (Powerline, Peach Innovation) records the components of the forces applied by the rowers to the oarlock along the *x*-axis (in the direction of the boat’s movement), the acceleration, the speed of the boat, and the angle formed by the oar with the *y*-axis (perpendicular to the boat’s movement).

A junior men’s coxless pair aged 17 years with 10 years’ experience in rowing participated in this study with the collaboration of their coach. The participants had no shared experience in rowing coxless pair together (i.e., this was their first season rowing together). The data collection occurred at the very first step of a 1-month crew-training period before the national championship in which the pair were to perform together. Both participants were current members of the French Rowing Academy (Nantes, France). The “stroke rower” is seated on the closest seat to the stern of the boat (i.e., he doesn’t see his teammate; see **Figure 1**) and, as described in the rowing training theories (Lippens, 2005), he propels the boat and set the rhythm. The “bow rower” is seated on the first seat, near to the bow of the boat (i.e., he sees the back of his teammate; see **Figure 1**) and he is supposed to follow the movement of the stroke rower to coordinate with him. Participants were in the top 10 of their category in France. This study was performed in accordance with the Declaration of Helsinki and the APA ethics guideline. It was approved by a local Institutional Review Board of the university. The two rowers and their coaches were informed of the procedures and gave their consent.

The two coxless rowers conducted a 12-min race of sub-maximal on-water rowing at 18–19 strokes per min (spm), as intended for the analysis. Sub-maximal is considered to be at 70–75% of the participant’s fastest speed, with a heart rate is below 145–156 beats per minute (bpm). This race thus account for the very first stage of team training, which was assumed to capture the initial learning processes of a newly formed crew composed of expert individual rowers.

### Data Collection

Two distinct data sets were collected to account for the activity of the two rowers during the race. First, the phenomenological data were recovered through individual self-confrontation interviews (Theureau, 2003) with each rower. Second, the behavioral data were recovered using an automatic mechanical device during the race.

#### Phenomenological Data Collection

The actors’ phenomenology was the starting point for the descriptions of the actor/environment coupling. This was consistent with the enactivist view to social couplings and the claim that human activity displays autonomous characteristics that are not reducible to behavioral descriptions (Varela et al., 1991). The enactivist approach therefore devotes special attention to pre-reflective self-conscious phenomena, that is, the implicit ways in which a given actor experiences his/her ongoing activity. To capture actors’ phenomenology through their pre-reflective self-consciousness embedded in the unfolding activity (i.e., lived experience), our study included a methodology that used phenomenological forms of retrospective interviews. From this perspective, at each instant of the race under study, we used self-confrontation interview techniques to collect the phenomenological data that accounted for the pre-reflective self-consciousness of the participants. This was consistent with recent enactive studies in sports (Bourbousson et al., 2011, 2012; Poizat et al., 2012; Sève et al., 2013; Bourbousson et al., 2015; Araujo and Bourbousson, 2016).

To this end, each rower of the coxless pair was filmed individually during the race by two video cameras located in a second boat that followed the rowing boat. Each rower was equipped with a high-fidelity microphone. Together, the recordings allowed us to collect the rowers’ behaviors and verbal communications. These behavioral traces of their activities helped us conduct the individual self-confrontation interviews immediately after the race. The self-confrontation interviews were designed so that rowers were asked to “re-experience their race” (i.e., re-enact their race) in order to describe and comment on the very details of the dynamics of their lived experience at each instant of the race (i.e., what they were doing, feeling, thinking, perceiving; see Theureau, 2003, for further details). Based on this verbalization data set, we were able to further characterize how the participants experienced each stroke. Each interview was fully recorded using a video camera so we able transcribe the verbal data and synchronize the rower’s verbalizations collected during the self-confrontation interview with the corresponding oar strokes. Each individual interview lasted 1 h.

#### Behavioral Data Collection

The behavioral data were obtained from collection of mechanical data during the race using the *Powerline* system (Peach Innovations, Cambridge, UK) at 50 Hz (Coker et al., 2009). This system is imperceptible to rowers, thus allowing them to perform in natural conditions, but in an instrumented boat. In line with the study’s aim, the system has a data acquisition and storage center connected to different sensors that allow to collect (a) the force applied to the oarlock by each rower (i.e., in the direction of the longitudinal axis of the boat), (b) the changes in each oar angle in the horizontal plane (i.e., the angle formed by the oar with the perpendicular axis to the longitudinal axis of the boat) and (c) the boat velocity and acceleration, via an accelerometer and a speed sensor fixed under the middle of the boat’s shell (see **Figure 1**). The accuracy of the force and angle sensors was 2% of full scale (1500 N) and 0.5°, respectively. The calibration of sensors was carefully checked before the experiment. The “drive” portion of a given stroke takes place in the water and propels the boat; it begins with a minimum oar angle (i.e., the catch) and ends with a maximum angle (i.e., the finish). Conversely, the “recovery” reflects the portion of the stroke that occurs out of water (Hill, 2002).

### Data Processing

#### Building the Individual Courses-of-Experience

To perform the empirical phenomenological description of the crew joint action, we mobilized the course-of-action framework. This framework is rooted in the enactivist approach, and it offers valuable analytic tools to operationalize the phenomenological claims of enactivism. Tightly linked to the phenomenology of Sartre (1958), the course-of-action analytical approach includes sophisticated accounts of the pre-reflective self-consciousness reported by the participants, allowing for a step-by-step analysis of the dynamics of the lived experience involved in the activity under study (Theureau, 2003).

In this light, verbalization data obtained from the interviews were fully transcribed and then synchronized in a **Table 1**. We then systematically reconstructed the ‘course-of-experience’ of each rower during the race from the verbalization data sets (Theureau, 2003) by identifying the chaining of phenomenological experiential units across time. A course-of-experience accounts for what is meaningful to the actor at each instant of the race. Phenomenological experiential units chained together over time thus provide a detailed description of the dynamics for a given actor. Considering the hypothesis of the course-of-action framework, a phenomenological experiential unit does not directly result from the verbalization data, but is built by the researcher based on this data. The researcher identifies the six components of each phenomenological experiential unit (i.e., the so-called hexadic sign, Theureau, 2003) that are assumed to merge at a given instant to form what the participant lives intrinsically as a syncretic experience. A given phenomenological experiential unit lasts until another unit begins from the point of view of the actor; its duration thus depends on the intrinsic sense-making dynamics of the rower. For instance, in the present study, the delineated units were close to the duration of an oar stroke (or shorter), reflecting the importance of each cycle in experiencing the race.

**Table 1:** Example of the synchronization of the rowers’ verbalization at the time code 00:40 [min:sec] of the race and their phenomenological experiential units filled regarding its six components.

Time code: 40 s	Stroke Rower			Bow Rower	
	Verbalization	Six components		Verbalization	Six components
*Rower 1:*	*Here, always good.*	**I**: To apply coach instructions/To hold on the back/Up hands on the front/To be synchronized with his partner/To have a maximum amplitude/To propel the boat. **E**: Be well synchronized with his partner/Row with a big amplitude/Maintain the boat pace. **K:** With the fatigue, he has trouble concentrating on the instructions. **P**: Coach instructions/feeling tired/Feel the boat as stable/Feel a good boat propulsion/Feel a well skiing of the shell. **A**: Feels the wind blowing on their back/Perceives the boat skiing. **RK**: *NI*	**Researcher**:	And now what about your feelings?	**I**: To drive well the boat/To keep the boat stable/To synchronize with his partner/Adapt to his partner/To put the same force as his partner during the oar stroke/To drag the boat as soon as possible/Be technically just in his movements **E**: Make a straight line with the boat/Be well synchronized with his partner/The boat should stay flat/Expects his partner grows as they usually do/Find ≪ the optimal intensity ≫ /Do not make technical fouls **K**: *NI* **P**: Feeling on the position of his body and its movements/sees the boat as stable **A**: Looks at the shoulders and hands of his partner/Puts on the same intensity as his partner/Perceives the boat skiing/Flexed his arm **RK**: Realizes that he made a technical error
**Researcher**:	Okay. Is this what you were thinking about at this moment? Or is it because you see the movie?		*Rower 2:*	*Here! We are already beginning to be a little more coordinated.*	
*Rower 1:*	*At this moment, I don’t think about this. I thought to propel more and I already began to feel the fatigue.*		**Researcher**:	And what about your technical point of view?	
**Researcher**:	Ah Yes …		*Rower 2:*	*I see that my outer arm is not necessarily stretched. Normally, this arm must be stretched, but I keep it a little bent.*	
*Rower 1:*	*Yes, so at this moment, I did not necessarily think to tell me… I felt as if the boat were skiing.*		**Researcher**:	Do you say it to yourself when you are rowing at this moment?	
**Researcher**:	Okay. But did you really feel at this moment the boat skiing?		*Rower 2*	*Yes*	
*Rower 1:*	*Yes, I felt the boat skiing on the water.*		**Researcher**:	Well …	
**Researcher**:	Okay.				

*I, Involvement in the situation; E, Expectations; K, prior mobilized Knowledge, P, Perception; A, Action; RK, Refashioned Knowledge. NI, not identified*

The first component of a phenomenological experiential unit refers to a current action [i.e., Action (A)], defined as the fraction of activity that the individual can show, tell, or comment on at a given moment. This component is the closest to the syncretic experience of the actor in the situation. It is assumed to emerge as a physical action, a communicative exchange, or an interpretative act. The researcher identified this component within the verbalization data sets by determining what the participant was doing and what he was thinking. The second component refers to the current involvement [i.e., Involvement in the situation (I)], defined as the individual’s concerns at a given moment. This component was identified within the verbalization data sets by identifying the participant’s significant concerns in relation to the specific situation. The third component refers to current expectations [i.e., Expectations (E)], defined as what is expected by the individual in the situation at a given moment. It was identified within the verbalization data sets by identifying the participant’s expectations about the current situation arising from his concerns and from the previous events in the setting (e.g., what result he/she was anticipating). The fourth component refers to knowledge elements [i.e., prior mobilized Knowledge (K)], defined as the individual’s past knowledge that is relevant to the current situation. This component was identified within the verbalization data sets by identifying the prior elements of knowledge used by the participant. The fifth component refers to the perception [i.e., Perception (P)], defined as elements of the situation significant to the individual at a given moment. It was identified within the verbalization data sets by identifying what the participant considered to be a meaningful element of the situation. The sixth component refers to the construction, validation, or invalidation of knowledge, defined as the component of activity that modifies elements of knowledge at a given moment [i.e., Refashioned Knowledge (RK)]. This component was identified within the verbalization data sets by identifying what knowledge was being constructed, validated, or invalidated by the participant at the considered instant. For further details on the method or the framework, see Theureau (2003). **Table 1** provides an example of each of these components [i.e., Action (A), Involvement in the situation (I), Expectations (E), prior mobilized Knowledge (K), Perception (P), and Refashioned Knowledge (RK)].

To enhance the coding process validity, the first, second, and the last author (who had already coded protocols of this type in earlier studies) randomly selected a 2-min sequence of activity for a crossed analysis. At this step, each researcher independently built the course-of-experience of each rower, and then compared their codes to identify disagreements. Any of these initial disagreements were resolved by discussion among the researchers, who debated their interpretations until a consensus was reached on the number of phenomenological experiential units and the contents of the six components of each unit. After this consensus was reached, the first author reconstructed the dynamics of the lived experience of each rower during the complete race. Remaining verbalization data that were doubtful or unclear were collectively re-processed. Then, the phenomenological experiential units were further aggregated to be processed through a thematic analysis of qualitative data (Braun and Clarke, 2006). The starting point of the thematic analysis was to characterize how each rower experienced each oar stroke in terms of joint action effectiveness (e.g., similarity of their sensation about the boat’s glide, or about their global perception about the boat/crew functioning). This characterization was based on in a detailed examination of the six components of each phenomenological experiential unit, so that the extent to which rowers experienced joint action effectiveness was identified by the researcher in a comprehensive analysis of each instant of the race (see Supplementary Image 1). Such an analysis allowed the researcher to decide how the rower experienced the joint action effectiveness, even if the rower was unable to detail such an experience in an explicit way. We were able to identify different individual typical modes of experiencing joint action effectiveness (i.e., from experiencing an effective to a detrimental joint action). From this local analysis, the first-order themes related to the joint action effectiveness experience were then merged step-by-step to give rise to second-order themes (see Braun and Clarke, 2006, for further details), which were the so-called typical modes of experiencing joint action effectiveness. Once these themes had been identified, each phenomenological experiential unit was labeled according to the theme to which it belonged, so that the chaining of the typical modes of experience might be analyzed across time.

After identifying and labeling the phenomenological experiential units, the next step consisted in time synchronizing the rowers’ typical experiences. Such synchronization allowed scrutinizing the extent to which rowers simultaneously and similarly experienced the effectiveness of their joint action during the ongoing performance. At this step, typical arrangements of the modes of experience were scrutinized which allowed us to delineate portions of joint action dynamics (i.e., phenomenological data samples) that were congruent (or not) with the related lived experiences. The aim of the following next step was to search for the mechanical signatures of such delineated phenomenological data samples.

#### Computing Mechanical Indicators at Various Levels of Description

Mechanical indicators were calculated for each rower’s stroke to account for individual-, interpersonal-, and boat-levels of description. These indicators were analyzed for each full oar stroke. Each stroke was decomposed into four phases to better assess changes within the mechanical signatures, specifically, the first and second halves of the drive phase (i.e., during the propulsive phase; when the oar was in water) and the first and second halves of the recovery phase (i.e., during the replacement phase; when the oar was out of water). Raw data (oar angles, forces applied to the oarlocks, acceleration and velocity) were filtered with a low pass Butterworth filter, with a 5 Hz cutoff frequency. Continuous angular velocities were then computed as the first derivative of the angular position, using the central difference formula. The continuous relative phase between oar angles of the stroke and the bow rower was selected to assess the interpersonal coordination (de Brouwer et al., 2013) and was calculated according to Hamill et al. (2000). Each cycle was considered between catch points as the local minimum of oar angle. Then, all the data were interpolated to 101 points per cycle. As the stroke rower’s cycle did not start at exactly the same time that the one of the bow rower’s, all studied rowing cycles were normalized on the stroke rower’s cycle of oar stroke in order to allow for the comparison between rowers.

##### Individual level of description

To account for the individual level of description of the mechanical parameters, 11 indicators were selected: (a) the mean of force applied by the rower to the pin of oarlock in the direction of the longitudinal axis of the boat (N), (b) the standard deviation of the force’s values (N), (c) the linear momentum of the force produced (kg.m.s^-1^), (d) the peak force (N), (e) the peak force’s timing in percentage of cycle (%), (f) the range of motion of the rowers (°), (g) the catch angle (°), (h) the mean of the angle of oar velocity (°.s^-1^), and (i) the mean of the standard deviation of the values of the oar’s angle of velocity (°.s^-1^). Individual parameters were selected from the literature of performance analysis in rowing (Kleshnev, 2011).

##### Interpersonal level of description

To analyze the mechanical parameters at an interpersonal level of description, seven indicators were retained, which all accounted for a degree of synchrony of the oars strokes: (a) the mean of the angle’s continuous relative phase (°), (b) the mean of the standard deviation of the angle’s continuous relative phase, (c) the gap between the timing of either catch angles (%), (d) the mean of the gap between each individual peak force level (N), and (e) the gap between the timing of each individual peak force (% of the cycle). These parameters were selected to account for the level of synchrony between the angles of the rowers (Williams, 1967; Lamb, 1989; Hill, 2002) and between the exerted forces (Schneider et al., 1978; Wing and Woodburn, 1995; Baudouin and Hawkins, 2004).

##### Boat level of description

To account for the boat’s level of description, two indicators were selected: the mean of the boat’s velocity (m.s^-1^) and the mean of the boat’s acceleration (m.s^-2^).

#### Identifying the Mechanical Signatures of the Typical Modes of Experience by a Subjectivity-Based Sampling Method

To combine phenomenological and behavioral data (i.e., typical modes of experiencing the race and the mechanical signatures at various levels of description), we performed a subjectivity-based sampling procedure. The procedure involved first scrutinizing the phenomenological data (i.e., the rowers’ course-of-experience) to delineate the samples of behavioral data to be compared (i.e., various ways of experiencing the strokes give rise to various delineated sections within the race that will be further processed/compared). Such a subjectivity-based sampling method has been well developed in enactivist neuroscience (e.g., Rodriguez et al., 1999; Lutz et al., 2002; Lutz and Thompson, 2003; Froese et al., 2014a,b). To our knowledge, this has not been used in the field of human movement or sports science. The principle is to guide the observational study (e.g., brain dynamics observation, behavioral dynamics observation) using phenomenological data collected during the same task. This procedure includes the human experience as a valuable facet of the activity under study and investigates the observational (i.e., behavioral) measures that contribute to their emergence.

To utilize this method, the time code of each typical mode of experience was recorded (i.e., starting/ending point of the given mode) to identify all intervals falling under the same typical mode of experience, subsequently, we aggregated them in a corresponding sample. Various samples of mechanical data were built from this procedure (i.e., respecting the time codes of the typical modes of experience), each of them thus reflecting different ways of experiencing the joint action. Each instant of the joint action (i.e., each cycle) was further characterized in terms of the similarity of the individual experiences of the rowers, using the three individual modes of effectiveness experiences captured during the thematic analysis of each participant’s activity. From the collective level of description of the lived experiences, we delineated four collective phenomenological categories (i.e., four samples) in our overall data set. Each of these categories comprised mechanical indicators measured for each cycle under consideration, resulting in multiple quantitative time series.

The first collective phenomenological category was labeled Simultaneously and Similarly Experienced as Meaningless (SSE-M). The second category was labeled Simultaneously and Similarly Experienced as Detrimental (SSE-D). The third category was labeled Simultaneously and Similarly Experienced as Effective (SSE-E). The fourth category was labeled Simultaneously Diverging Experiences (SDE).

Statistical analysis was carried out on the mechanical signatures of each of the four categories using the SPSS 17.0 statistical software package (SPSS, Inc., Chicago, IL, USA). Descriptive statistics were reported using the mean and the standard deviation (mean ± SD). Differences between the four categories regarding each mechanical indicator were analyzed using a multivariate analysis of variance (MANOVA). When the main effect was significant, ANOVA, or two-way ANOVA, with Tukey’s HSD *post hoc* test was applied to the categories (SSE-M, SSE-D, SSE-E, and SDE) and the rowers (Rower 1 and Rower 2) as independent variables and the mechanical indicators listed above as dependent variables. *Post hoc* analyses were applied with Bonferroni correction. Data and ANOVA residuals were checked carefully for normal distribution using QQ plots. When distributions were not normal, a Kruskal–Wallis test was applied. When the Kruskal–Wallis tests were applied and revealed significant effects, Dunn’s tests was applied, as *Post hoc* analyses, to identify the location of differences between categories (Dunn, 1961). The level of significance was set at *p* < 0.05.

## Results

### Typical Individual Rowers’ Modes of Experiencing the Joint Action Effectiveness

The thematic analysis performed on the individuals’ phenomenological data showed that three main themes (i.e., revealing typical modes of experience) fit the collected data, suggesting three related recurrent ways of experiencing joint action effectiveness from the individual rowers’ points of view. The most prevalent typical mode of experience (75.5% of the time of their individual activities) accounted for the units of experience in which the joint action was experienced as “meaningless” by the rower. “Meaningless” was used here as a label to signify that the rower did not pay attention to the joint action at the pre-reflective level of their activity. The second and the third typical modes of experience accounted for the units of experience in which the participant reported a “salient” experience of the joint action. Especially, the second typical mode (16.3% of the time of their individual activities) accounted for portions of activity in which the rower reported a salient, meaningful experience of contributing to an effective oar stroke, indicating that the joint action was experienced as being particularly “effective.” The third typical mode of experience (8.2% of the time of their individual activities) accounted for portions of activity in which the rower reported a salient, meaningful experience of contributing to a poor oar stroke, thus indicating that the joint action was experienced as being “detrimental.”

### Collective Phenomenological Categories and Their Prevalence

**FIGURE 2:**
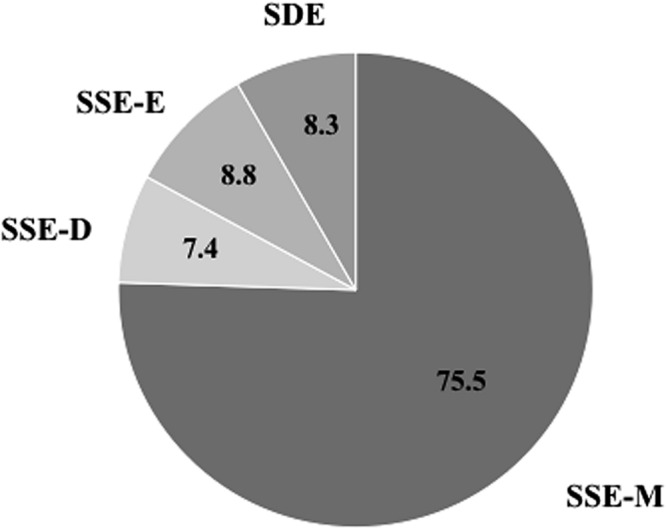
**Representation of the prevalence of the collective phenomenological categories during the race under study (in percentages).** The percentage is the ratio between the number of cycle in each collective phenomenological category and the total number of cycle recorded during the race. SSE-M, Joint action Simultaneously and Similarly Experienced as Meaningless; SSE-D, Joint action Simultaneously and Similarly Experienced as Detrimental; SSE-E, Joint action Simultaneously and Similarly Experienced as Effective; SDE, Simultaneously Diverging Experiences of joint action.

The first collective phenomenological category was built by aggregating the data related to all cycles (i.e., oar strokes) that the participants simultaneously and similarly experienced as being “meaningless” (*N* = 154 cycles out of 204 cycles, representing 75.5% of the race). This category was labeled SSE-M. The second category accounted for all cycles that the participants simultaneously and similarly experienced as being “detrimental” for the joint action (labeled SSE-D; *N* = 15 cycles; representing 7.4% of the race). The third category accounted for all cycles that were simultaneously and similarly experienced by the participants as being “effective” for the joint action (labeled SSE-E; *N* = 18 cycles; representing 8.8% of the race). The fourth category accounted for all cycles that the rowers simultaneously experienced in a diverging fashion, and it was labeled SDE (N = 17 cycles; representing 8.3% of the race). See the illustration in **Figure 2**.

### Mechanical Signatures of the Collective Phenomenological Categories at Three Levels of Analysis

The mechanical parameters related to the four identified categories (SSE-M, SSE-D, SSE-E, and SDE) were then submitted for further statistical analysis. The analyses aimed to identify the level of organization of the joint action (i.e., individual, interpersonal, or boat-level of the mechanical parameters analysis) that could at best explain the differences in the four collective phenomenological categories. For all of the following analyses, the comparison between the categories considered seven ways of analyzing the cycles: (a) the full cycle, (b) the drive phase, (c) the first half of the drive, (d) the second half of the drive, (e) the full recovery phase, (f) the first half of the recovery, and (g) the second half of the recovery.

#### Individual Level of Analysis

At the individual level of analysis, no significant differences between collective phenomenological categories was found in terms of individual mechanical indicators. The following indicators were assessed and did not capture differences between the categories: the mean force applied by the rower on the pin of the oarlock, the standard deviation of the force’s values, the linear momentum of the force produced, the peak force level, the peak force’s timing in the percentage of cycle, the range of motion of the rowers, the mean of the angle of oar velocity, the mean of the standard deviation of the values of the oar’s angle of velocity. See Supplementary Tables S1–S3.

#### Interpersonal Level of Analysis

**FIGURE 3:**
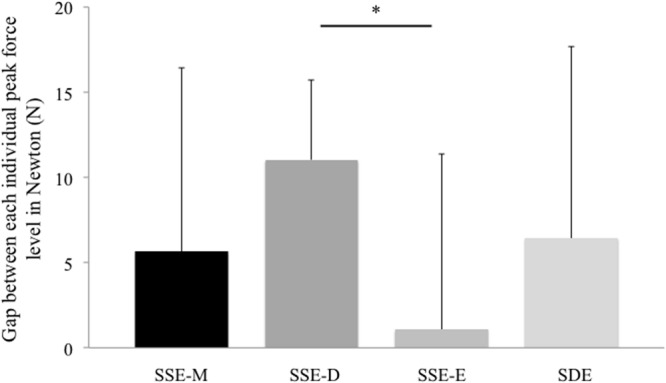
**Mean values and standard deviations for the gap between each individual peak force level in each identified subjectivity-based sample.** The gap was calculated by subtracting the peak force level of the bow rower with the peak force level of the stroke rower (i.e., a mean near 0 or negative is closest to the expert pattern). SSE-M, Joint action Simultaneously and Similarly Experienced as Meaningless; SSE-D, Joint action Simultaneously and Similarly Experienced as Detrimental; SSE-E, Joint action Simultaneously and Similarly Experienced as Effective; SDE, Simultaneously Diverging Experiences of joint action. Statistical significance was set to ^∗^*P* < 0.05

At the interpersonal level of analysis, the values of the relative phase measures did not differ significantly between categories. The main result at this level of analysis was related to the measure of the gap between their peak force levels, which was significantly higher for the SSE-D than for the other collective phenomenological categories. Indeed, the Kruskal–Wallis test revealed an effect between the categories (chi-squared = 8.451; df = 3; *p*-value = 0.038), and Dunn’s test then showed a significant difference between the SSE-D and the SSE-E categories (adjusted *p*-value = 0.026; see **Figure 3**). Thus, the measure of the gap between each individual peak force level appeared to be the best candidate to understand the mechanical parameters that supported a shared experience of effectiveness in joint action. See Supplementary Table S4.

#### Boat Level of Analysis

At the level of analysis of the boat, the results did not show differences between the four collective phenomenological categories for either of the indicators related to the boat, which were the mean of the boat’s velocity and the boat’s acceleration. See Supplementary Table S5.

## Discussion

The objective of the present study was to understand how individual experts in rowing experienced the effectiveness of their joint action when they rowed together at the first stage of their team coordination learning process. To achieve this objective, we collected the data related to their real-time lived experience (i.e., at a pre-reflective level of the activity) and to the related mechanical properties during a 12-min race. We were thus able to explore the mechanical signatures of various shared lived experiences. The discussion of the results is organized around our research questions. The results first suggested that (a) the extent to which rowers simultaneously experienced salient, meaningful sensations of effectiveness (i.e., effective or detrimental) in their joint action correlated with the extent to which supporting a mechanical signature captured expert-like pattern of team coordination. Secondly, the results also pointed out that (b) the participants spent a large amount of their activity not having a salient, meaningful experience of their joint action. These results are discussed regarding inter- and extra-personal regulation processes, respectively. We conclude by discussing the heuristics of an enactivist approach to social coupling in sports science.

### The Mechanical Signatures of the Salient, Joint Experiences of Effectiveness

When considering instances in which both rowers simultaneously and similarly had salient experiences of their joint action at a given instant, we obtained two samples that reflected the identified collective phenomenological categories, and that consisted of the measured mechanical parameters. The first collective phenomenological category accounted for simultaneous salient experiences of an effective joint action, the second for a salient shared experience of a detrimental joint action. The comparison of both collective phenomenological categories showed significant differences within their mechanical signatures. On the one hand, the pattern of the joint action that was Simultaneously and Similarly Experienced as Effective (SSE-E) showed that both rowers produced their peak force at the same time and peak force levels were very close. On the other hand, the pattern of the joint action Simultaneously and Similarly Experienced as Detrimental (SSE-D) revealed that both rowers produced their peak force in the same time, but their peak force diverged in terms of level: the peak force of the bow rower was higher than the one of the stroke rower. Interestingly and consistent with what the rowing literature describes in terms of what is expected of a rowing crew coordination (Smith and Draper, 2002; Baudouin and Hawkins, 2004), the mechanical pattern related to the shared experience of effectiveness (SSE-E category) was more expert than the pattern related to the shared experience of a detrimental joint action (SSE-D category). Indeed, coxless pair-oar rowing requires a high technical level as the force pattern required is more complex than in other rowing boats (Smith and Draper, 2002): it requires a specific pattern of application of the force due to the position of the two rowers in the boat. In order to maintain the boat’s direction, the stroke rower has to produce his peak force slightly earlier than the bow rower does and with a peak force higher than that the bow rower (Smith and Draper, 2002; Baudouin and Hawkins, 2004). In this light, our results showed that the gap between the peak force level of the stroke and the bow rower was significantly more important when the rowers similarly experienced their joint action as detrimental, but this gap was inverted compared to expert patterns (i.e., bow rower’s peak force level was higher than that the one of the stroke rower). Joint sense making thus appeared to be nested in the behavioral facet of the joint action in that the extent to which rowers shared experience of effectiveness was related to the extent to which their mechanical patterns signed expert team rowing.

**FIGURE 4:**
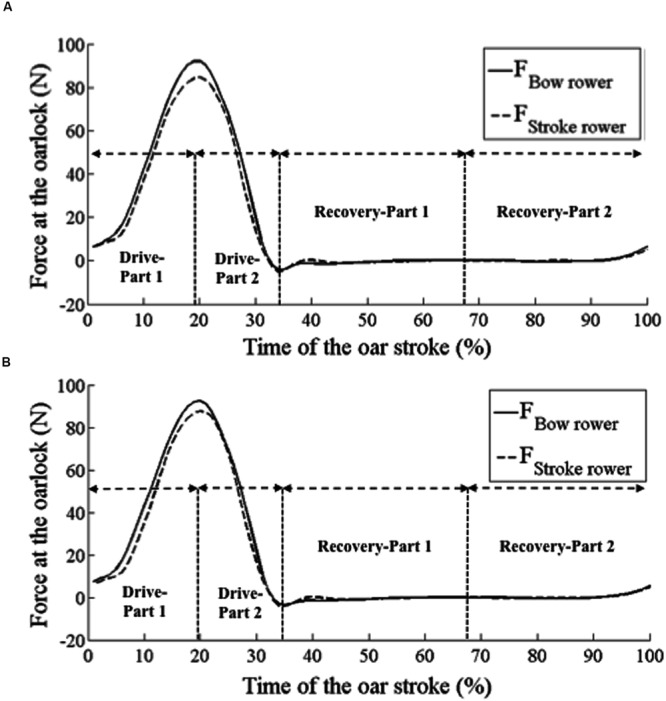
**(A)** Representation of the force mean at the oarlock time curve of a typical oar stroke experienced as detrimental and **(B)** representation of the force mean at the oarlock time curve of a typical oar stroke experienced as effective for bow and stroke rowers.

Moreover, while experiences of joint action were quite accurate in terms of the mechanical states from which they emerged (see the Section “Results” discussed above) our results pointed out that these experiences were still capable of improvement. Indeed, at a pre-reflective level of the activity, the rowers did not perceive that their joint action states were not perfectly achieved in terms of what is expected for a coxless pair crew. Additional mechanical indices supported this interpretation: the analysis of the rower’s peak force showed that this peak was produced a little bit late by the stroke rower (around 1%; see **Figure 4**), as required in the rowing literature (Smith and Draper, 2002; Baudouin and Hawkins, 2004). This finding implies that rowers were not fully aware of the team coordination patterns that shaped their joint action and supported their lived experiences, suggesting that future research should address avenues related to the unperceived features of team coordination phenomena (e.g., Varlet and Richardson, 2015), especially how these features can change through training practice.

In sum, the gap in the rowers’ peak force levels shaped the emerging shared experiences of effectiveness, which indicates that the *interpersonal* level of mechanical description was the one that best accounted for the extent to which rowers experienced their joint action effectiveness at the pre-reflective level of their activity. Interestingly, by pointing out that rowers managed the continuity/change of their joint action from the interpersonal states of coordination they perceived, our study indicates that an “inter-personal” regulation mode might structure how each rower manages the joint action. The Inter-personal mode of regulation refers to individual activities that are synchronized through informational constraints relied on by the given actors. For example, this mode of regulation is implied in studies where participants are asked to synchronize their oscillating limbs and to actively regulate the emergent states of coordination on the basis of the extent of synchrony they perceive on-the-fly. Such inter-personal regulation processes have been investigated in lab-based studies regarding inter-arms coordination between participants (Davis et al., 2016), for inter-legs synchronization (Schmidt and Richardson, 2008), or in natural settings regarding inter-oars’ stroke coordination in rowing (Wing and Woodburn, 1995) or inter-players’ trajectories coordination in basketball (Esteves et al., 2011). In terms of the experience that each actor has in his actor–environment coupling, such a regulation mode assumes that actors remain sensitive to the dynamic behavior of the partner, and that they adapt in this regard, as found in the present study from the analysis of the salient, meaningful shared experience of joint action effectiveness. The following “Results and Discussion” Sections will counterbalance such a conclusion by suggesting that “extra-personal” modes of regulation might have shaped some remaining portions of the race (i.e., SSE-M category).

While having an individual salient, meaningful experience of effectiveness in a joint action did not guaranteed that this lived experience was similar to that of the teammate or that it was related to expert-like mechanical signatures, our results supported the idea that when an experience was shared, it was likely to emerge from an efficient joint action. However, there was a notable size difference between experiential categories (e.g., between SSE-M and the other experiential categories). This difference in the size of the collective phenomenological categories could be the reason it was difficult to obtain significant results at the mechanical level.

### Participants Did Not Make an Extensive Salient Experience of Their Joint Action

**FIGURE 5:**
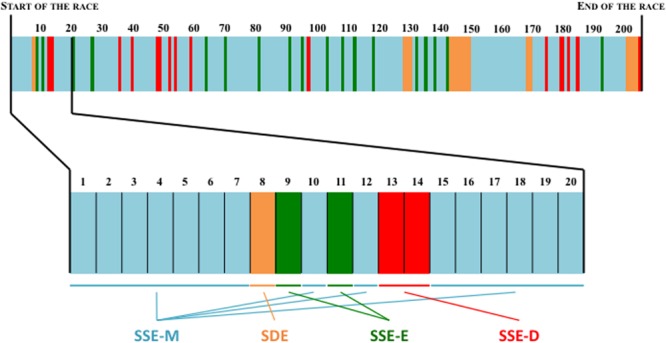
**Overview of the repartition of the collective level of description of the lived experiences throughout the race**.

Beyond the analysis focused on the shared salient experiences of different degrees of effectiveness, the analysis of the phenomenological data provides elements to counterbalance the “inter-personal” mode of regulation suggested above. To this end, the prevalence of each typical mode of experiencing the joint action (i.e., each collective phenomenological category) needs to be considered. Joint action was perceived simultaneously as a salient, meaningful experience for only 24,5% of the race under study. With respect for this typical mode of experiencing joint action as salient, of note is that 8.3% of which was associated with diverging experiential content (i.e., the joint action’s degree of effectiveness was simultaneous and salient but not similarly experienced), and 16.2% with similar experiential contents. In the latter case, the rowers could simultaneously and similarly report a salient, meaningful experience of a given stroke as effective or detrimental to their joint action (i.e., SSE-E and SSE-D categories), as extensively discussed in the previous section. Finally, the results showed that, at the pre-reflective level of their activity, the rowers did not pay attention to the effectiveness of their joint action for the remaining 75.5% of the studied period, indicating that the rowers did not make an extensive salient experience of their joint action at the scale of the overall race (see the distribution of the collective phenomenological categories during the race in **Figure 5**). In other words, and as labeled in the thematic analysis of the phenomenological data, the rowers were able to coordinate their strokes through experiencing their joint action as “meaningless” during a large part of their crew activity.

Thus highlighting that the joint action generally was not explicitly lived as a salient experience within the dynamics of the rowers’ activity might be considered unexpected. Indeed, by revealing the prevalence of implicit team processes at the early stages of a team coordination learning process, this result is controversial in that it does not support the implicit coordination process hypothesized by Eccles and Tenenbaum (2004) in sports team learning, which viewed the learning of team coordination as a linear process progressing from explicit processes toward implicit and embodied processes. At least, our present findings suggest that team coordination in rowing seems to be a task, which can be performed by individual experts in rowing without their exhibiting an extensive intentional/explicit activity of co-regulation of their joint action.

With respect to the discussion about the modes of regulation that underlie the present joint action (e.g., an “inter-personal” mode of regulation), and in indicating that interpersonal states of coordination were not the constant focus of the adaptations actively performed by the rowers, our observations now suggest that extra-personal regulation processes might also have underlain the joint action dynamics (Millar et al., 2013). Extra-personal regulation has been used to explain the emergence of team coordination patterns while rowers were only regulating their individual coupling to the environment separately. The environment is thus used by individuals to mediate/organize the arrangement of individual activities at each moment of the collective activity. This process differs from inter-personal regulation processes that are grounded on a direct co-regulation of the joint action dynamics itself. When a rower is involved in an extra-personal regulation and acts on his/her oar, he/she can adjust his/her movements in response to the reaction of the water and the boat information. Both rowers can thus respond similarly, thanks to this mediation. Interestingly, as observed within social insects that act together through environmental mediation (e.g., termites, ants), such a process does not need individual agents to be aware of the collective motion to which they are contributing, which might thus explain the very few instances in the present study where the rowers made salient, meaningful experiences of their joint action.

**FIGURE 6:**
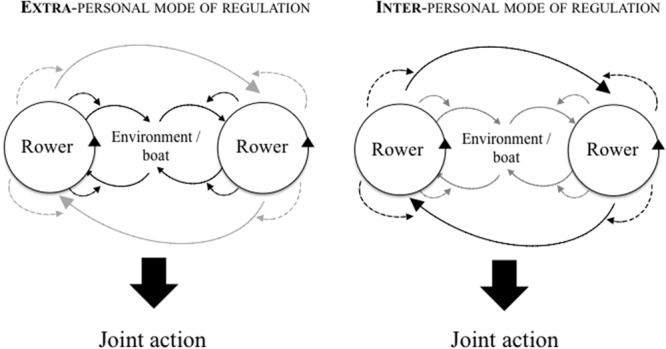
**Illustration of both of the suggested modes of regulation used by the rowers (From an initial representation proposed by Froese and Di Paolo, 2011)**.

Remembering that the analysis of the shared salient experiences of effective/detrimental oar strokes suggested that the rowers’ regulation processes were rooted in the inter-personal level of organization, the additional finding notes that the rowers did not make an extensive experience of their joint action for a large part of the race. This finding thus suggests that the rowers used an extra-personal regulation process to regulate their coordination in the portions of the race when joint action was experienced as “meaningless.” One can then question how the extra-personal regulation process suggested here can combine with the inter-personal mode of regulation captured earlier. We assume that, when rowing alone, expert rowers learn to regulate their activity through the boat’s information (Millar et al., 2013), which allow them to row with others using an extra-personal mode of regulation, even if they have no prior shared team practice. However, along the coordination process under study, some events occurred at the inter-personal level of organization (i.e., synchronization breakdowns) to which the rowers were sensitive, causing them to exhibit an inter-personal mode of regulation at the level of the activity that was salient and meaningful to them (see **Figure 6** for an illustration of the two suggested modes of regulation). This latter mode, even being less prevalent, might be the mode that they use to manage their progression, the mode they use to maintain/change the flow of their joint action effectiveness. Thus, to hypothesize what might be observed later in the future stages of learning their joint action, two alternative transformations might be evidenced: (a) rowers will increase the proportion of cycles lived as “meaningless” (i.e., SSE-M), thus signing an increasing extra-personal mode of regulation of their continuous joint action. At the same time, they maintain an inter-personal mode of regulation to manage race events, evidenced through momentary salient experiences of joint action effectiveness that is rooted in interpersonal mechanical states; or (b) rowers will also gradually learn to regulate the sudden events through an extra-personal mode of regulation, evidenced through salient experiences of joint action effectiveness that is rooted in the boat’s mechanical variation. In this light, future research should investigate the extent to which rowers are supposed to share more salient meaningful experience through team training, considering the nature and the transformation of the information that support such experiences. Such research should be able to better challenge the Eccles and Tenenbaum’s (2004) hypothesis that assumes a hypothetical pathway from explicit to implicit regulation processes in team coordination learning.

Interestingly, our study also revealed that when rowers simultaneously experienced a salient joint action, their experience was not necessarily similar. However, such dissimilarities in the simultaneous experiential content did not appear to link with any decrement in the mechanical measures. Our suggestion is that, as long as rowers experienced simultaneously their co-regulation of the joint action, the joint performance did not suffer from each rower judging effectiveness differently. This corroborates that team coordination patterns of movement may occur without a perfectly shared experience about the ongoing joint action (Bourbousson et al., 2011, 2012). Other studies have indicated that joint action was quite resilient to perfectly shared experiences, especially in those that used the perceptual crossing paradigm (Auvray et al., 2009). This device puts two actors in situations where they have to move an avatar in a virtual environment populated by different entities (avatars of humans and various lures), visually empty but providing tactile stimulation at each encounter through the mouse used by the participants. Interestingly, what helps participants to experience social connectedness, and subsequently to succeed in finding each other, is the ongoing co-regulation process they both perceived simultaneously (Froese et al., 2014a), disregarding the extent to which each actor was satisfied by the unfolding interaction, since they have no feedback on their current effectiveness in the task. In agreement with the findings obtained in such experimental studies, the present study provided further evidence that the full coordination of sense-making activities is not needed to allow for a viable patterned joint action in a natural task, as long as actors are simultaneously involved in co-regulating their collective behavior (Froese and Di Paolo, 2011; Froese et al., 2014a,b). Thus, as recently introduced as a hot topic in sports team coordination research (Araujo and Bourbousson, 2016), future research should consider the ways in which the extrinsic facet of the coordination process (e.g., the behavioral facet) and the phenomenological facet are mutually constrained to give rise to collective effectiveness in a task. Regarding training concerns, future research should consider how shared repeated practice of joint action (i.e., through the development of team coordination expertise) might change step-by-step the relationships that shape both facets.

#### Insights into Team Coordination Phenomena in General

Beyond our hypotheses, the results of the present study offer some insights into team coordination phenomena in general. First, the team members combined two ways of regulating their joint action throughout the race, namely a meaningless regulation and a salient, meaningful regulation of the joint action. While such a distinction has been proposed by Eccles and Tenenbaum (2004) in their framework for team coordination in sports, related research questions remain open to understand the effectiveness of such regulation processes, as illustrated by the present results, which challenge this theory. Second, the team members also combined two distinct modes of regulation, inter- and extra-personal. While such a distinction has been suggested in human movement science (e.g., Millar et al., 2013), very little is known about how both modes of regulation might co-occur during a given ongoing joint action.

Considered together, these distinct regulation processes call for three main avenues in team coordination research. Firstly, research should question the settings’ characteristics that are particularly propitious for one of these processes. For instance, the environmental mediation possibilities might call for a prevalence of extra-personal regulation. Also, the number of participants involved in the collective behavior might make the inter-personal regulation process hard to manage (i.e., each participant cannot regulate all the dyadic linkages included in the collective), so that extra-personal processes might become parsimonious and preferable when environmental mediation is available. Secondly, research should question to what extent training practices could change regulation, and for which benefits such transformations might occur. Thirdly, research might identify the parameters that control how actors switch dynamically from one regulation process to another during an unfolding joint action.

Beyond the need for team coordination research not only to focus on the behavioral facet of the joint effort, but also to investigate the underlying modes of intentional regulation, our opinion is that future avenues will benefit from considering hypotheses included in the stigmergic theory of collective behavior (Susi and Ziemke, 2001; Avvenuti et al., 2013) in which holistic phenomena of coordination might be considered as emerging from the behavior–environment coupling. Stigmergic theory of collective behavior explains how each agent of the social system regulates its own behavior–environment coupling, without the agents needing to actively and directly coordinate with other agents, and without them needing to be aware of these cooperating agents. Of interest is that no evidence of such processes has been discussed extensively in human collective behavior. The scarce references made to such collective behaviors (e.g., Bourbousson et al., 2012; Silva et al., 2014) have neglected the stigmergic hypothesis and instead have adopted the local-and-distributed mode of coordination, i.e., humans can exhibit a patterned collective behavior without needing to grasp the global properties of the social structure to which they contribute. When considering that stigmergic processes do not require the actors to be aware of the collective behavior (e.g., like social insects that do not experience a sense of working together), then stigmergic processes could explain why in this study, the rowers were in synch for the three quarters of the race without simultaneously having a salient, meaningful experience of their joint action effectiveness. For instance, when the extra-personal mode of regulation (i.e., stigmergic) is needed to become an expert crew in rowing, it also seems to operate easily in a novice crew (despite their intentional subjective regulation being shaped by inter-personal processes). Such stigmergic processes could also explain why the rowing training theory (Morrow, 2011) does not consider the step-by-step adjustments of team coordination as a time consuming part of the training. It could also explain why rowing crews are often composed late in the sporting season, because of members’ interchangeability are facilitated when actors coordinate through the environment (in comparison to the increased member-dependence obtained through inter-personal regulation processes). At least, the present study suggests ways for future research to delineate strengths and weaknesses of the regulatory activities that facilitate the emergence of collective behavioral patterns.

#### The Heuristics of an Enactivist Approach to Social Couplings

Beyond our hypotheses and methodological aims, the results of the present study provide the opportunity to explore the potential of the enactivist approach to social couplings (Laroche et al., 2014; Araujo and Bourbousson, 2016). We believe the approach offers benefits to research in this area. First, this framework is constructivist, linked to a dynamic approach to behavior and to an additional phenomenological epistemology (Thompson, 2011). The framework is concerned with combining an understanding of team coordination from an external point of view (i.e., mechanical measures) with an understanding of the (inter)subjectivity that shapes/is shaped by this behavioral facet (Petitmengin and Bitbol, 2009). The phenomenological assumptions included in this framework were thus useful for capturing in detail the actors’ experiences at each instant of the joint action. By comparing the individual situated experiences of the rowers, the researchers were able to characterize the dynamic properties of team members’ participation in joint sense making (De Jaegher and Di Paolo, 2007) and the specific timing and sequencing of such lived experiences. As performed in the present study, this subjectivity-based description guided the subsequent processing of the behavioral data. In this light, we used the lived experiences of rowers to delineate various collective phenomenological categories and the related behavioral samples sets that were then compared statistically. This procedure, inspired by works conducted in the area of enactivist neurosciences (e.g., Lutz et al., 2002), has been referred to as a subjectivity-based sampling method.

The subjectivity-based sampling method provided three opportunities. It allowed us to process quantitative and behavioral data only, which *de facto* included the phenomenology that prevailed in such data. However, as is usual in behavioral research, the processed team coordination data (i.e., the explained variable) include mostly the experimental condition, specifically external/contextual constraints that have been observed (i.e., the explanatory variables). The procedure performed in our study guided the mechanical analysis of the phenomenological data, and thus ideally illustrated how a full enactivist approach could be used with behavioral data. The subjectivity-based sampling appeared to be a good method for interdisciplinary research. Moreover, by including a phenomenological methodology that uses retrospective interview techniques, the research design permits activity to be studied based on the reconstruction of the natural and specific conditions of the activity to reveal how participatory sense-making develops in a real-world setting. Finally, the present method instantiates the concept of human movement as a place of interplay of behavioral and phenomenological facets (Froese and Di Paolo, 2010), and a concept of team coordination as a simultaneous combination of the behavioral dynamics of a joint effort (i.e., non-accidental correlations between the movements of the participants) and participatory sense-making dynamics (i.e., each participant constraining the own-world of the other). The present study illustrates the interiority of individuals that is not always captured by objectivist approaches. Here, taking into account lived experiences helped to make sense of variability in the objective data, and illustrated how this interiority might be the starting point to describe actor/environment coupling, including actor/actor coupling. However, the subjectivity-based sampling method should be strengthened by future research in order to better identify its domain of relevance, that is the particular setting in which an elicitation of actors’ lived experiences heuristically complements behavioral analyses.

## Conclusion

Our study leaves some open questions. In this study, extra-personal regulation processes have been suggested for most of the race, but instances of intentional inter-personal regulation processes might also be suggested, as similar salient, meaningful lived experiences of joint action effectiveness were explained by mechanical parameters, accounting for an inter-personal level of organization. Further research could be conducted with the same methodology (a) to extend the heuristics of a subjectivity-based sampling method and (b) to address the question of dynamic changes in the intentional modes of regulation during races or in more advanced training sessions. When one assumes that rowers learn to be an expert team by actively regulating and coordinating their activity based on what they experience as being effective, then one can question how such behavioral changes may occur without rowers having a pervasive lived experience of their joint efforts. Thus, a promising question may be to focus on team coordination training, first, by addressing how such a practice may progressively change the saliency of the participants’ lived experiences of joint action and second, by addressing how it may change the behavioral signatures in which those lived experiences are anchored. Together, these questions of interest suggest that integrating lived experience with the investigation of joint action is likely to improve our understanding of how actors regulate their interaction in real time to facilitate stable and optimal forms of social functioning.

## Author Contributions

MR’K and JB have made substantial, direct and intellectual contribution to the work, and approved it for publication. JS and MD made substantial contributions to the analysis and the interpretation of the data. They also gave final approval of the version to be published.

## Conflict of Interest Statement

The authors declare that the research was conducted in the absence of any commercial or financial relationships that could be construed as a potential conflict of interest.
