# Supplementary material for: Joint Action of a Pair of Rowers in a Race: Shared Experiences of Effectiveness Are Shaped by Interpersonal Mechanical States
Source: Front Psychol. 2016 May 18;7:720. doi: 10.3389/fpsyg.2016.00720 (PMC4870391; doi:10.3389/fpsyg.2016.00720)
Supplement: Supplementary file 1 [file Table_1.PDF]

Supplementary Table 1. Indices' mean for the full cycle. The four subjectivity-based samples identified in the phenomenological analysis are distinguished regarding individual level of description of the mechanical parameters.

	**SSE-M** *(N=154)*				**SSE-D** *(N=15)*				**SSE-E** *(N=18)*				**SDE** *(N=17)*			
	Stroke Rower		*Bow Rower*		*Stroke Rower*		*Bow Rower*		*Stroke Rower*		*Bow Rower*		*Stroke Rower*		*Bow Rower*	
	Mean	*SD*	*Mean*	*SD*	*Mean*	*SD*	*Mean*	*SD*	*Mean*	*SD*	*Mean*	*SD*	*Mean*	*SD*	*Mean*	*SD*
Force at oarlock (N)	14.33	1.19	15.75	1.16	13.97	0.65	15.68	1.02	14.35	1.24	15.65	1.50	14.13	0.67	15.69	1.09
SD Force (N)	27.47	1.92	29.30	2.25	26.94	1.39	29.46	2.29	27.68	1.71	29.25	2.73	27.06	1.19	29.18	2.28
Linear momentum (kg.m.s^{-1})	52.93	4.84	48.19	4.96	47.22	2.33	53.04	4.42	48.10	3.63	52.58	5.70	47.48	2.11	52.72	3.75
Force peak (N)	87.03	6.46	92.50	8.60	84.49	6.13	95.52	6.46	90.21	4.13	91.73	9.58	85.52	4.67	91.95	8.60
Force peak timing (%)	19.09	1.89	18.69	1.53	18.73	1.10	18.67	1.11	19.28	1.27	18.50	1.50	18.71	1.45	18.59	1.18
Range of motion (°)	88.67	1.51	88.32	1.43	88.61	1.98	88.38	0.96	89.06	1.56	88.64	1.68	89.23	1.65	88.87	1.16
Catch Angle (°)	-53.17	1.38	-57.55	1.21	-53.07	1.03	-57.28	1.34	-53.47	1.72	-57.81	0.92	-53.66	1.01	-57.77	1.02
Angle oar velocity (°.s-1)	-0.02	0.54	0.04	0.63	0.04	0.65	-0.33	0.80	0.03	0.57	0.06	0.69	-0.06	0.49	-0.07	0.52
SD Angle oar velocity (°.s-1)	60.10	2.29	59.38	2.18	59.80	1.73	59.50	1.11	60.25	1.98	59.64	1.27	60.30	1.71	59.90	1.16
