# Supplementary material for: Joint Action of a Pair of Rowers in a Race: Shared Experiences of Effectiveness Are Shaped by Interpersonal Mechanical States
Source: Front Psychol. 2016 May 18;7:720. doi: 10.3389/fpsyg.2016.00720 (PMC4870391; doi:10.3389/fpsyg.2016.00720)
Supplement: Supplementary file 3 [file Table_3.PDF]

Supplementary Table 3. Indices' mean for each part of the recovery phase. The four subjectivity-based samples identified in the phenomenological analysis are distinguished regarding individual level of description of the mechanical parameters.

	**SSE-M** *(N=154)*				**SSE-D** *(N=15)*				**SSE-E** *(N=18)*				**SDE** *(N=17)*			
	Stroke Rower		*Bow Rower*		*Stroke Rower*		*Bow Rower*		*Stroke Rower*		*Bow Rower*		*Stroke Rower*		*Bow Rower*	
	Mean	*SD*	*Mean*	*SD*	*Mean*	*SD*	*Mean*	*SD*	*Mean*	*SD*	*Mean*	*SD*	*Mean*	*SD*	*Mean*	*SD*
	**The Recovery phase**															
Force at oarlock (N)	-0.12	0.26	-0.10	0.26	-0.06	0.24	-0.01	0.24	-0.06	0.21	-0.20	0.28	-0.05	0.21	-0.04	0.30
SD Force (N)	1.61	0.41	1.72	0.43	1.60	0.42	1.79	0.53	1.62	0.51	1.75	0.35	1.59	0.33	1.67	0.43
Angular amplitude (°)	87.95	1.78	87.64	1.42	87.56	2.67	87.82	1.11	88.22	1.83	87.94	1.88	88.61	1.93	88.38	1.14
Angular velocity ($°.s^{-1}$)	-40.19	2.97	-38.60	1.96	-39.55	3.39	-38.09	2.04	-40.14	2.81	-38.12	1.75	-41.20	3.48	-39.34	1.57
SD Velocity ($°.s^{-1}$)	15.62	2.85	10.47	2.37	15.60	3.75	10.89	3.49	15.42	3.07	11.14	1.05	15.42	3.03	9.88	2.57
	**First half of the Recovery**															
Force at oarlock (N)	-0.74	0.36	-0.77	0.35	-0.65	0.35	-0.74	0.30	-0.68	0.37	-0.80	0.33	-0.63	0.26	-0.67	0.33
SD Force (N)	1.31	0.71	1.15	0.45	1.32	0.63	1.18	0.45	1.36	0.68	1.08	0.34	1.17	0.59	0.93	0.31
Angular amplitude (°)	39.16	3.26	39.91	2.19	38.80	3.24	41.37	2.19	39.26	2.97	39.62	1.98	40.26	3.44	40.74	2.12
Angular velocity ($°.s^{-1}$)	-35.59	4.59	-34.85	2.91	-34.81	4.64	-34.72	3.26	-35.51	4.08	-34.88	2.80	-37.24	5.27	-36.09	2.80
SD Velocity ($°.s^{-1}$)	-35.59	4.59	-34.85	2.91	17.73	4.94	10.25	4.94	17.64	3.67	9.60	2.78	17.21	4.47	9.03	3.79
	**Second half of the Recovery**															
Force at oarlock (N)	0.50	0.28	0.59	0.37	0.54	0.27	0.74	0.46	0.56	0.20	0.60	0.46	0.53	0.26	0.59	0.42
SD Power (W)	70.54	12.26	70.99	11.56	70.99	11.88	69.46	14.20	67.98	13.05	71.33	12.87	76.69	12.88	73.97	10.52
Angular amplitude (°)	48.79	3.03	47.74	2.12	48.76	2.99	46.45	2.33	48.96	3.16	48.32	1.59	48.35	2.87	47.64	1.97
Angular velocity ($°.s^{-1}$)	-44.84	3.18	-42.35	2.53	-44.34	3.57	-41.49	2.52	-44.83	3.36	-42.83	2.50	-45.16	3.05	-42.58	2.01
SD Velocity ($°.s^{-1}$)	10.75	2.21	9.17	2.22	10.35	3.43	9.80	2.27	10.32	2.61	8.70	1.85	11.18	2.93	8.95	2.26
