# Supplementary material for: Joint Action of a Pair of Rowers in a Race: Shared Experiences of Effectiveness Are Shaped by Interpersonal Mechanical States
Source: Front Psychol. 2016 May 18;7:720. doi: 10.3389/fpsyg.2016.00720 (PMC4870391; doi:10.3389/fpsyg.2016.00720)
Supplement: Supplementary file 4 [file Table_4.PDF]

Supplementary Table 4. Indices' mean for each part of the cycle. The four subjectivity-based samples identified in the phenomenological analysis are distinguished regarding interpersonal level of description of the mechanical parameters.

	**SSE-M** *(N=154)*		**SSE-D** *(N=15)*		**SSE-E** *(N=18)*		**SDE** *(N=17)*	
	Mean	*SD*	*Mean*	*SD*	*Mean*	*SD*	*Mean*	*SD*
	**The full cycle**							
Difference timing catch angle (s)	0.05	0.04	0.07	0.04	0.06	0.04	0.06	0.04
Angle CRP (°)	2.21	2.68	2.57	3.37	1.54	2.71	2.00	2.46
SD Angle CRP (°)	9.90	2.20	10.40	2.39	8.91	1.90	9.79	2.72
Gap between each force peak level (N)	5.64	10.78	11.03	4.69	1.06	10.33	6.43	11.21
Gap timing between each force peak (%)	0.36	1.48	0.07	0.88	0.78	1.63	0.12	1.50
	**The Drive phase**							
Angle CRP (°)	4.66	7.78	2.85	9.59	4.89	7.64	2.78	5.72
SD Angle CRP (°)	11.87	4.15	11.74	3.10	10.45	4.00	11.26	4.23
	**First half of the Drive**							
Angle CRP (°)	6.50	10.54	5.31	11.32	8.26	11.19	3.25	8.95
SD Angle CRP (°)	11.50	4.91	11.22	4.83	9.75	4.69	10.13	3.60
	**Second half of the Drive**							
Angle CRP (°)	8.57	3.76	0.39	9.31	1.50	5.82	2.26	5.49
SD Angle CRP (°)	8.93	5.30	9.25	4.45	7.52	3.56	9.15	6.66
	**The Recovery phase**							
Angle CRP (°)	-13.08	44.55	-15.91	40.45	-24.12	38.14	-12.79	55.16
SD Angle CRP (°)	92.61	32.44	88.43	32.76	95.93	25.28	97.60	29.65
	**First half of the Recovery**							
Angle CRP (°)	-10.46	23.46	-7.60	18.53	-16.03	22.59	-11.71	25.25
SD Angle CRP (°)	27.36	11.54	21.66	7.17	29.75	13.98	27.17	13.64
	**Second half of the Recovery**							
Angle CRP (°)	-15.95	71.49	-24.33	70.26	-32.30	59.89	-14.32	88.08
SD Angle CRP (°)	121.17	45.18	115.18	44.09	127.63	33.68	127.74	38.02

Note: CRP= Continuous Relative Phase
