# Supplementary material for: Joint Action of a Pair of Rowers in a Race: Shared Experiences of Effectiveness Are Shaped by Interpersonal Mechanical States
Source: Front Psychol. 2016 May 18;7:720. doi: 10.3389/fpsyg.2016.00720 (PMC4870391; doi:10.3389/fpsyg.2016.00720)
Supplement: Supplementary file 5 [file Table_5.PDF]

Supplementary Table 5. Indices' mean for each part of the cycle. The four subjectivity-based samples identified in the phenomenological analysis are distinguished regarding boat level of description of the mechanical parameters.

	**SSE-M** *(N=154)*		**SSE-D** *(N=15)*		**SSE-E** *(N=18)*		**SDE** *(N=17)*	
	Mean	*SD*	*Mean*	*SD*	*Mean*	*SD*	*Mean*	*SD*
	**The full cycle**							
Boat velocity ($m.s^{-1}$)	3.38	0.17	3.41	0.06	3.41	0.06	3.39	0.05
Boat acceleration ($m.s^{-2}$)	0.03	0.06	-0.002	0.07	0.01	0.04	0.01	0.05
	**The Drive phase**							
Boat velocity ($m.s^{-1}$)	2.85	0.20	2.88	0.10	2.87	0.08	2.89	0.11
Boat acceleration ($m.s^{-2}$)	0.69	0.13	0.64	0.13	0.66	0.09	0.68	0.12
	**First half of the Drive**							
Boat velocity ($m.s^{-1}$)	2.38	0.21	2.43	0.06	2.42	0.06	2.39	0.06
Boat acceleration ($m.s^{-2}$)	-0.53	0.39	-0.66	0.41	-0.63	0.43	-0.40	0.46
	**Second half of the Drive**							
Boat velocity ($m.s^{-1}$)	3.30	0.22	3.31	0.17	3.31	0.14	3.37	0.20
Boat acceleration ($m.s^{-2}$)	1.90	0.35	1.93	0.42	1.93	0.38	1.76	0.45
	**The Recovery phase**							
Boat velocity ($m.s^{-1}$)	3.67	0.14	3.69	0.07	3.70	0.06	3.66	0.07
Boat acceleration ($m.s^{-2}$)	-0.33	0.07	-0.34	0.05	-0.33	0.06	-0.36	0.05
	**First half of the Recovery**							
Boat velocity ($m.s^{-1}$)	3.79	0.15	3.83	0.09	3.82	0.08	3.79	0.07
Boat acceleration ($m.s^{-2}$)	-0.14	0.04	-0.13	0.05	-0.15	0.04	-0.16	0.04
	**Second half of the Recovery**							
Boat velocity ($m.s^{-1}$)	3.55	0.13	3.56	0.07	3.58	0.07	3.53	0.08
Boat acceleration ($m.s^{-2}$)	-0.50	0.12	-0.54	0.13	-0.50	0.11	-0.56	0.10
