# Supplementary material for: Joint Action of a Pair of Rowers in a Race: Shared Experiences of Effectiveness Are Shaped by Interpersonal Mechanical States
Source: Front Psychol. 2016 May 18;7:720. doi: 10.3389/fpsyg.2016.00720 (PMC4870391; doi:10.3389/fpsyg.2016.00720)
Supplement: Supplementary file 6 [file Image_1.PDF]

Supplementary Image 1. Illustration of how the collective phenomenological categories were obtained. At the step of identifying the components of the phenomenological experiential units, words in grey are components that remain active at the considered instant, but which were delineated through front units to the current unit of experience. Words in black highlight the components that were especially identified through the present verbalizations.

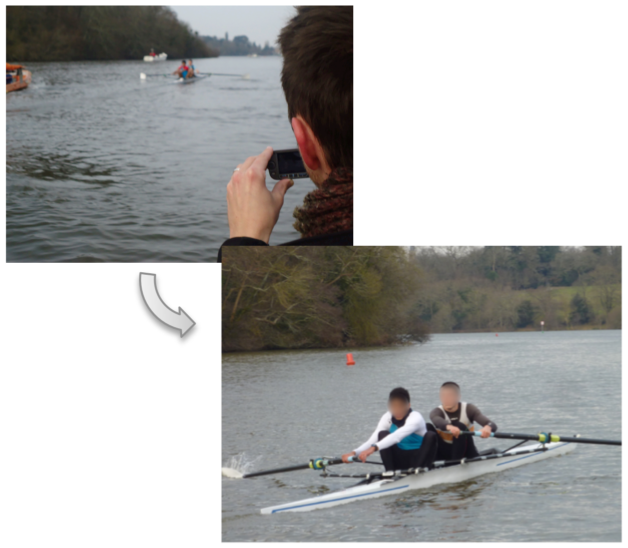

Video recording of the crew activity

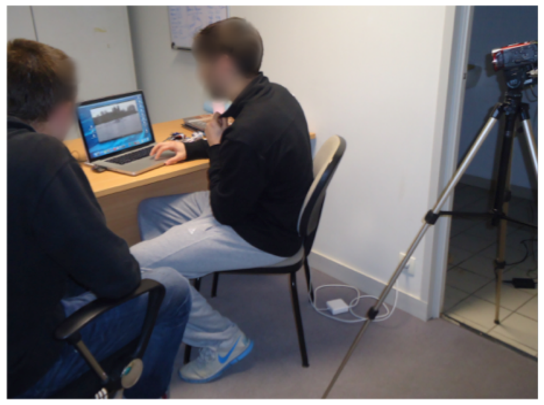

Individual self-confrontation interview

DATA COLLECTION (DEDICATED TO THE PHENOMENOLOGICAL ANALYSIS)

VERBALIZATION

Time code: (min: sec)

00:40

Stroke Rower

Rower 1:	*Here, always good*
**Researcher:**	Okay. Is this what you were thinking about at this moment? Or is it because you see the movie?
Rower 1:	*At this moment, I don't think about this. I thought to propel more and I already began to feel the fatigue.*
**Researcher:**	Ah Yes …
Rower 1:	*Yes, so at this moment, I did not necessarily think to tell me… I felt as if the boat were skiing*
**Researcher:**	Okay. But did you really feel at this moment the boat skiing?
Rower 1:	*Yes, I felt the boat skiing on the water.*
**Researcher:**	Okay.

00:45

Bow Rower

**Researcher:**	And now what about your feelings?
Rower 1:	*Here ! We are already beginning to be a little more coordinated.*
**Researcher:**	And what about your technical point of view?
Rower 1:	*I see that my outer arm is not necessarily stretched. Normally, this arm must be stretched, but I keep it a little bent.*
**Researcher:**	Do you say it to yourself when you are rowing at this moment?
Rower 1:	*Yes*
**Researcher:**	Well …

PHENOMENOLOGICAL EXPERIENTIAL UNIT NUMBER: 11

Stroke Rower:

I: To apply coach instructions / To hold on the back / Up hands on the front / To be synchronized with his partner / To have a maximum amplitude / To propel the boat

E: Be well synchronized with his partner / Row with a big amplitude / Maintain the boat rhythm

K: With the fatigue, he has trouble concentrating on the instructions

P: Coach instructions / feeling tired / Feel the boat as stable / Feel a good boat propulsion / Feel a well skiing of the shell

A: Feels the wind blowing on their back / Perceives the boat skiing

RK: *NI*

Bow Rower:

I: To drive well the boat / To keep the boat stable / To synchronize with his partner /Adapt to his partner / To put the same force as his partner during the oar stroke / To drag the boat as soon as possible / Be technically just in his movements

E: Make a straight line with the boat / Be well synchronized with his partner / The boat should stay flat / Expects his partner grows as they usually do / Find « the optimal intensity » / Do not make technical fouls

K: *NI*

P: Feeling on the position of his body and its movements / sees the boat as stable

A: Looks at the shoulders and hands of his partner / Puts on the same intensity as his partner / Perceives the boat skiing / Flexed his arm

RK: Realizes that he made a technical error

THEMATIC ANALYSIS

Joint action experienced as "**Effective**"

Joint action experienced as "**Effective**"

COLLECTIVE PHENOMENOLOGICAL CATEGORY

Simultaneously and Similarly Experienced as Effective (SSE-E)

Note: I= Involvement in the situation, E= Expectations, K= prior mobilized Knowledge, P= Perception, A= Action, RK= Refashioned Knowledge
